# Metabolic Syndrome and Risk of Upper Tract Urothelial Carcinoma: A Case-Control Study From Surveillance, Epidemiology and End Results-Medicare-Linked Database

**DOI:** 10.3389/fonc.2020.613366

**Published:** 2021-01-21

**Authors:** Yi Lu, Wei Zhang, Shujun Fan, Zhen Liang, Zhongjia Li, Jia Tian, Jiaqi Kang, Yuxuan Song, Kang Liu, Kechong Zhou, Xiao Wang, Yongjiao Yang, Xiaoqiang Liu

**Affiliations:** ^1^ Department of Urology, Tianjin Medical University General Hospital, Tianjin, China; ^2^ Division of Epidemiology, School of Medicine, University of California, Riverside, CA, United States; ^3^ Department of Urology, The Second Hospital of Tianjin Medical University, Tianjin, China

**Keywords:** metabolic syndrome, Surveillance, Epidemiology and End Results (SEER)-Medicare, upper tract urothelial carcinoma, case-control study, incidence

## Abstract

**Background:**

Metabolic syndrome (MetS) and its components are associated with increased risks of several cancers. However, the relationship between MetS and upper tract urothelial carcinoma (UTUC) has never been investigated before.

**Methods:**

We identified 3,785 UTUC cases aged over 65 years old within the Surveillance, Epidemiology and End Results-Medicare database between 2007 and 2016. For comparison, non-cancer controls (n = 189,953) were selected from the 5% random sample of individuals residing within regions of SEER registries and matched with cases through diagnosis date and pseudo-diagnosis date. MetS and its components were all defined by using ICD-9-CM codes. Multivariate logistic regression models were conducted to calculate odds ratios (ORs) and 95% confidence intervals (CIs). Time trends for MetS and its components were reported and we also performed dose-response effect analysis to test the concomitant effect of these components. The study was presented following the STROBE reporting checklist.

**Results:**

UTUC risk was associated with metabolic syndrome (NCEP-III: OR: 1.669, 95% CI: 1.550–1.792; IDF: OR: 1.924, 95% CI: 1.676–2.172) and its component factors: elevated waist circumference/central adiposity (OR: 1.872, 95% CI: 1.693–2.055), impaired fasting glucose (OR: 1.306, 95% CI: 1.133–1.480), high blood pressure (OR: 1.295, 95% CI: 1.239–1.353), high triglycerides (OR: 1.280, 95% CI: 1.222–1.341), and low high-density lipoprotein cholesterol (OR: 1.354, 95% CI: 1.118–1.592). Consistent associations could also be observed in the subgroup analyses by tumor stages, grades, and tumor size. Additionally, the rates of MetS increased over time in both UTUC and control cohort (NCEP-III criterion; EAPC: +18.1%, *P <*0.001; EAPC: +16.1%, *P <*0.001, respectively). A significantly gradual increase in UTUC rates could be seen as the No. of the MetS components increase (*χ*² = 37.239, *P*_trend_ = 0.000).

**Conclusions:**

Among people aged over 65, MetS and its components were significant risk factors for UTUC with consistent associations in different tumor stages, grades, and tumor size. Even if a subject who did not meet the criteria for MetS had only one of the components, he (she) still had an elevated risk for UTUC. Strategies to control the epidemic of MetS and its components might contribute to a reduction in the UTUC burden. The findings should be considered tentative until ascertained by more researches.

## Introduction

Upper tract urothelial carcinoma (UTUC), the transitional cell carcinoma of the ureter or renal pelvis, is a type of relatively rare tumor, which occurs in approximately two people per 100,000 population in the world (1). Currently, radical nephroureterectomy (RNU) is the standard treatment for localized UTUC, while the survival outcomes after the surgery are far from satisfactory (1). Moreover, around 56% of UTUC cases are locally advanced or muscle-invasive at presentation because of its occult symptoms and delayed diagnosis, which results in poor prognosis and less available treatments (2). Both inherited and environmental factors, such as tobacco use, aromatic amines exposure play crucial roles in the pathogenesis of UTUC and these factors have been developed to assist with the risk stratification of UTUC (3–5). However, more valuable preoperative factors and the mechanisms of UTUC initiation and development remain to be investigated and clarified.

The metabolic syndrome (MetS) is composed of a cluster of metabolic risk factors including elevated waist circumference/central adiposity, impaired fasting glucose, high blood pressure, and dyslipidemia. It is widely believed that all of the factors and MetS are commonly associated with cardiovascular disease, type 2 diabetes and according to the American Heart Association/National Heart, Lung, and Blood Institute, there are as many as 35% of Americans have MetS (6). Nowadays, cumulating evidence has indicated the associations between MetS and several types of cancers (7), such as prostate cancer (8), colorectal cancer (9), hepatocellular carcinoma (10), and bladder cancer (11). Emerging evidence has also demonstrated the prognostic value of MetS and its components in several cancers (12, 13). Nevertheless, researches regarding the role of MetS in UTUC, which shares similar risk factors with bladder cancer are still lacking and it remains to be investigated whether MetS and its components are risk factors of UTUC.

In response to the gap in this field, we utilized data from the Surveillance, Epidemiology and End Results (SEER)-Medicare linked database, which links insurance claims data to state cancer registry data. The large sample size allowed us to illustrate the independent role of MetS and each component of MetS on UTUC. Additionally, we were also able to evaluate the associations between MetS and pathological characteristics of UTUC, including tumor stage, grade, and tumor size, which have been shown to affect the outcomes in other types of cancers (10, 11, 14, 15). The following article was presented in accordance with the STROBE reporting checklist.

## Methods

### Study Design and Data Source

We utilized the Surveillance, Epidemiology and End Results (SEER) Medicare linked Database to define a case-control study. Cases were identified through the SEER registries and exposure records were ascertained *via* the SEER-Medicare linked data. The SEER database, which covers approximately 28% of the US population is a cancer database including data on patients’ clinical demographics, clinical and pathological characteristics, treatment information, and cause of death. The SEER-Medicare database links Medicare is the federal U.S. health insurance program for elderly adults aged 65 and older and is the primary health insurer for about 97% of individuals aged ≥65 years in the U.S. Because of the great samples in the database, it can be representative of the general population (16). In the study, we recorded the following items: demographics, comorbidity information, tumor features, and drug use.

### Study Population

Using the International Classification of Diseases for Oncology, Third Edition (ICD-O-3), we identified all subjects diagnosed with UTUC (renal pelvis, or ureter. Site codes 65.9, and 66.9, respectively) in the SEER-Medicare linked database between 2007 and 2016 (n = 13,587). The SEER-Medicare data are currently available up to 2016, we thus selected a most recent decade period. A comparison group of controls with no prior cancer diagnoses was selected from the 5% random sample of individuals residing within regions of SEER registries. Cases were excluded for the following reasons: two or more cancer diagnosis (n = 2,327), enrollment in a Medicare health maintenance organization (HMO) plan within 3 years of diagnosis and absence of both Medicare parts A and B (n = 5,630) (This resulted in a minimum age of 68 years for the study participants), age at diagnosis less than 65 years old and enrolled in Medicare for reasons other than age (n = 675), diagnosis only from autopsy/death certificate (n = 143), missing information on month of diagnosis (n = 347), and any unknown or non-available information (n = 680). Notably, patients who were diagnosed as bladder cancer and validated to be recurrent from UTUC were also included (n = 419). Medicare covers a few individuals aged <65, the cutoff value for age (≥65) thus was chosen to reduce selection bias. The final cohort included 3,785 UTUC patients. The control selection was based on the same inclusion and exclusion criteria as the case selection. 189,953 controls were finally included in the study. By using a random number generator, each control was assigned a pseudo-diagnosis date. Cases and controls were matched by diagnosis date and pseudo-diagnosis date to minimize the effect of possible diagnostic trends.

### Definition of Metabolic Syndrome, Metabolic Component Conditions, and Covariates

The International Classification of Diseases revision 9 (ICD-9-CM) code and corresponding medical conditions that were used to define MetS are provided in **Supplementary Table 1**. Notably, before 2001, there was no available specific ICD-9-CM code for elevated waist circumference and central adiposity. From 2001 to 2016, the code for central adiposity was included in the definition, therefore we only included the record of elevated waist circumference/central adiposity according to the diagnostic criteria. Although the relatively low frequency of low high-density lipoprotein (HDL) cholesterol, the condition was also included in the analysis to evaluate whether it was an independent risk factor for UTUC and it was also included in the definition of MetS. In the study, MetS was defined based on two definitions. The first, according to the US National Cholesterol Education Program Adult Treatment Panel III (NCEP-III), MetS was defined as the presence of three or more of the following conditions: central adiposity or elevated waist circumference, impaired fasting glucose (including type II diabetes), high triglycerides, low HDL cholesterol, and hypertension, based on the ICD-9-CM and Current Procedural Terminology (CPT)-4 codes summarized in **Supplementary Table 1**. The second, according to the International Diabetes Foundation (IDF), MetS was defined as the presence of central adiposity/elevated waist circumference and any two of the other factors: impaired fasting glucose (including type II diabetes), hypertension, low HDL cholesterol, and high triglycerides. The following risk factors: tobacco smoking (yes or no) (codes: V15.82, 989.84, 305.1, respectively) and alcohol use (yes or no) (codes: 303, 305.0, V11.3, V79.1, 291) were also extracted. All these codes used in the study had been well described in previous studies (10, 11).

### Charlson Comorbidity Index Score

According to a previously validated method (17), the CCI score was derived from the Medicare claims data and was categorized into low (0–2), moderate (3–5), and severe (≥6).

### Statistical Analysis

All the qualitative data was presented as frequency (proportions). All the quantitative data was shown in mean ± standard deviation (SD). *χ*² test and independent Student’s *t*-test were used to compare categorical data and numerical data, respectively. Estimated annual percentage changes (EAPC), calculated by using a generalized linear model, was tested to show temporal trends of MetS and individual MetS components and utilized Venn diagrams to reveal the distributions of MetS and its components in both UTUC and the control cohort. We calculated adjusted odds ratios (ORs) and 95% confidence intervals (95% CIs) to evaluate the association between MetS and the components of MetS and the risk of UTUC by using logistic regression models. The same methods were also adopted in the dose-response effect analysis and the evaluation of MetS and its potentially related factors. The multivariable logistic regression models included the following adjustment factors: time of diagnosis/matching date, age at diagnosis (continuous), gender (male, female), race (White, Black, Asian, Hispanic, other), registry area, household income, drug use, Medicare/Medicaid dual enrollment, time of enrollment in Medicare, and tobacco and alcohol use. Furthermore, we also evaluated the association between MetS and its component factors with UTUC stages (pT and pN), UTUC grades, and tumor size by conducting logistic regression models comparing the UTUC subgroups to the entire control group. We also conducted multivariate analyses comparing UTUC subgroups within the UTUC cohort to assess the relationships between MetS and its components and UTUC severity. A two-tailed *P* < 0.05 was regarded statistically significant in all data analyses. All analyses were performed using STATA 12.0 (Stata-Corp.).

## Results

Detailed records of the characteristics of the study population were summarized in **Table 1**. The majority of cases and controls between 2007 and 2016 were white, male, non-alcohol users, and non-smokers. There were no significant differences in terms of age at diagnosis, gender, race, registry area, household income, Medicare/Medicaid dual enrollment, and time of enrollment in Medicare between cases and controls (*P* > 0.05 for all). UTUC cases had greater percentages of smoking, alcohol use, and higher CCI score compared with the controls (*P*<0.05 for all). Among UTUC cases, the prevalence of MetS components ranged from 1.5% for low HDL cholesterol to 65.7% for high blood pressure. Among the controls, the prevalence of MetS components ranged from 1.1% for low HDL cholesterol to 61.6% for high blood pressure. The prevalence of MetS was 17.1% (NCEP-III) and 5.3% (IDF), and 10.4% (NCEP-III) and 2.8% (IDF) in cases and controls, respectively (**Supplementary Figures 1** and **2**). UTUC cases had significantly higher proportions of both MetS components and MetS than the controls (*P*<0.05 for all). Time trends analysis demonstrated an increase in the rates of MetS patients (NCEP-III criterion; UTUC: EAPC: +18.1%, *P* < 0.001; Controls: EAPC: +16.1%, *P* < 0.001). Similarly, the rates of impaired fasting glucose (UTUC: EAPC: +1.2%, *P* < 0.001; Controls: EAPC: +0.9%, *P* < 0.001), high blood pressure (UTUC: EAPC: +1.4%, *P* < 0.001; Controls: EAPC: +1.5%, *P* < 0.001), elevated waist circumference/central adiposity (UTUC: EAPC: +14.4%, *P* < 0.001; Controls: EAPC: +8.0%, *P* < 0.001), high triglycerides (UTUC: EAPC: +21.1%, *P* < 0.001; Controls: EAPC: +23.1%, *P* < 0.001), and low HDL cholesterol (UTUC: EAPC: +0.11%, *P* < 0.001; Controls: EAPC: +0.09%, *P* < 0.001) also increased over time in the two cohorts (**Supplementary Figures 3** and **4**).

**Table 1:** Characteristics of UTUC cases and controls, SEER-Medicare (2007–2016).

Characteristics	UTUC cases (n = 3,785)		Controls (n = 189,953)		*P*-value^§^
	n	%	n	%	
Mean age, years (SD)	76.0 (6.4)		75.9 (5.7)		0.286
Gender					0.314
Female	1423	37.6	72942	38.4	
Male	2362	62.4	117011	61.6	
Ethnicity					0.255
White	3289	86.9	166019	87.4	
Black	238	6.3	11397	6.0	
Asian	107	2.8	5509	2.9	
Hispanic	87	2.3	4179	2.2	
Other	64	1.7	2849	1.5	
Resgistry site					0.089
San Francisco	174	4.6	9118	4.8	
Connecticut	348	9.2	17286	9.1	
Detroit	371	9.8	19375	10.2	
Hawaii	42	1.1	2849	1.5	
Iowa	394	10.4	18235	9.6	
New Mexico	110	2.9	5889	3.1	
Seattle	276	7.3	13107	6.9	
Utah	121	3.2	5699	3.0	
Atlanta	144	3.8	7028	3.7	
San Jose	106	2.8	5319	2.8	
Los Angeles	303	8.0	14816	7.8	
Rural Georgia	<11^‡^	–	1330	0.7	
Greater California	435	11.5	20515	10.8	
Kentucky	238	6.3	12727	6.7	
Louisiana	182	4.8	9498	5.0	
New Jersey	534	14.1	27163	14.3	
Median household income, $					0.681
≤35,000	950	25.1	47489	25.0	
35,001– 60,000	1927	50.9	95737	50.4	
>60,000	908	24.0	46727	24.6	
Time of enrollment in Medicare, years					0.684
3–5	871	23.0	43689	23.0	
5–11	1919	50.7	97446	51.3	
≥ 11	995	26.3	48818	25.7	
Medicare/Medicaid dual enrollment	655	17.3	33052	17.4	0.850
Tobacco use (Yes)	1154	30.5	45969	24.2	0.000
Alcohol use (Yes)	182	4.8	2469	1.3	0.000
Charlson comorbidity score					0.000
Low (0–2)	3509	92.7	172667	90.9	
Moderate (3–5)	242	6.4	15766	8.3	
Severe (≥6)	34	0.9	1520	0.8	
Tumor location					
Renal pelvis	1856	49.0			
Ureter	1929	51.0			
AJCC T stage					
pTis,a	1041	27.5			
pT1	893	23.6			
pT2	606	16.0			
pT3,4	1245	32.9			
AJCC N stage					
pN0	3395	89.7			
pN+	390	10.3			
AJCC M stage					
pM0	3516	92.9			
pM1	269	7.1			
Tumor grade					
Low	1730	45.7			
High	2055	54.3			
Tumor size, cm					
≤3	2239	59.2			
>3	1546	40.8			
Metabolic conditions					
Impaired fasting glucose	1037	27.4	39700	20.9	0.000
High blood pressure	2487	65.7	117011	61.6	0.000
elevated waist circumference/ central adiposity	254	6.7	6648	3.5	0.000
Low HDL cholesterol	57	1.5	2089	1.1	0.018
High triglycerides	1446	38.2	57556	30.3	0.000
Metabolic syndrome					0.000
NCEP-III	647	17.1	19755	10.4	0.000
IDF	201	5.3	5319	2.8	0.000

^§^Comparison between UTUC cases and the controls.

^‡^Cell sizes less than 35 (race) and less than 11 (location) are not shown following the SEER-Medicare data use agreement.

**Table 2** presented the associations between UTUC and MetS components as well as MetS (defined by the NCEP-III and the IDF). Results of the multiple logistic analysis showed that elevated waist circumference/central adiposity, captured from the medical records, was associated with an almost double risk of UTUC compared with the controls (OR: 1.872, 95% CI: 1.693–2.055). It can also be observed that impaired fasting glucose (OR: 1.306, 95% CI: 1.133–1.480), low HDL cholesterol (OR: 1.354, 95% CI: 1.118–1.592), high blood pressure (OR: 1.295, 95% CI: 1.239–1.353), and high triglycerides (OR: 1.280, 95% CI: 1.222–1.341) were also associated with increased risk of UTUC. In the study, MetS, defined by NCEP-III and IDF criteria, was associated with an increased risk of UTUC (NCEP-III: OR: 1.669, 95% CI: 1.550–1.792; IDF: OR: 1.924, 95% CI: 1.676–2.172). Notably, we also tested the dose-response effect through multivariable logistic regression analysis (**Supplementary Table 2**). Results showed a significantly gradual increase in the strength of the association between UTUC rates and No. of the MetS components when 1 (OR: 1.600, 95% CI: 1.437–1.782), 2 (OR: 2.152, 95% CI: 1.715–2.590), 3 (OR: 2.530, 95% CI: 2.218–2.886), 4 (OR: 2.844, 95% CI: 2.455–3.335), or 5 (OR: 4.292, 95% CI: 3.166–5.418) MetS components were recorded in each individual patient, respectively (*χ*² = 37.239, *P*_trend_ = 0.000).

**Table 2:** Adjusted ORs and 95% CIs for the association between MetS and its components and UTUC.

	UTUC cases (n = 3,785)	Controls (n = 189,953)	OR (95% CI)^a^
	*N*	*N*	
Metabolic conditions			
Impaired fasting glucose	1037	39700	1.306 (1.133, 1.480)
High blood pressure	2487	117011	1.295 (1.239, 1.353)
elevated waist circumference/central adiposity	254	6648	1.872 (1.693, 2.055)
Low HDL cholesterol	57	2089	1.354 (1.118, 1.592)
High triglycerides	1446	57556	1.280 (1.222, 1.341)
Metabolic syndrome			
NCEP-III	647	19755	1.669 (1.550, 1.792)
IDF	201	5319	1.924 (1.676, 2.172)

^a^ORs adjusted for time of diagnosis date, age at diagnosis, gender, race, registry area, household income, drug use, Medicare/Medicaid dual enrollment, time of enrollment in Medicare, tobacco and alcohol use.

The results of the association between MetS and its components and risk of UTUC by different tumor grades were shown in **Table 3**. As we can see, all the components of MetS were significantly associated with increased risks of both low-grade UTUC and high-grade UTUC (ORs ranged from 1.014 to 2.375). MetS, defined by both NCEP-III and IDF standard, was also associated with increased risk for both low-grade UTUC and high-grade UTUC (ORs ranged from 1.383 to 2.368). Based on the results of multivariate analysis within the UTUC cohort, there were no differences in the MetS and its components-tumor grade associations.

**Table 3:** Adjusted ORs and 95% CIs for the association between MetS and its components and tumor grade, SEER-Medicare.

	High grade (n = 2,055)		OR (95% CI)^a,‡^	Low grade (n = 1,730)		OR (95% CI)^a,‡^	OR (95% CI)^a,†^
	n	%		n	%		
Metabolic conditions							
Impaired fasting glucose	557	27.1	1.235 (1.143, 1.337)	480	27.8	1.327 (1.228, 1.436)	0.967 (0.839–1.117)
High blood pressure	1354	65.9	1.147 (1.101, 1.190)	1133	65.5	1.062 (1.018, 1.104)	1.018 (0.890–1.165)
elevated waist circumference/central adiposity	148	7.2	1.992 (1.673, 2.375)	106	6.2	1.656 (1.349, 1.963)	1.188 (0.918–1.537)
Low HDL cholesterol	29	1.4	1.382 (1.014, 1.714)	28	1.7	1.580 (1.126, 2.033)	0.808 (0.483–1.356)
High triglycerides	804	39.1	1.261 (1.185, 1.334)	642	37.1	1.226 (1.143, 1.308)	1.087 (0.954–1.240)
Metabolic syndrome							
NCEP-III	358	17.4	1.658 (1.507, 1.827)	289	16.7	1.615 (1.442, 1.788)	1.045 (0.881–1.240)
IDF	111	5.4	1.832 (1.503, 2.237)	90	5.2	1.742 (1.383, 2.102)	1.051 (0.790–1.401)

^a^ORs adjusted for time of diagnosis date, age at diagnosis, gender, race, registry area, household income, drug use, Medicare/Medicaid dual enrollment, time of enrollment in Medicare, tobacco and alcohol use.

^†^Comparison between high-grade UTUC and low-grade UTUC in the UTUC cohort.

^‡^Comparison between control group and high-grade UTUC or low-grade UTUC.

**Table 4** presented the association between MetS and its components and the risk of UTUC by different tumor pT stages. It could be observed that all the components of MetS were significantly associated with increased risks of both locally advanced T stage (T3-T4) UTUC and non-advanced T stage UTUC (ORs ranged from 1.006 to 2.578), which was also observed in MetS, defined by both NCEP-III and IDF criteria (ORs ranged from 1.478 to 2.494). Results of the multivariate analysis within the UTUC cohort showed that there were no differences in the MetS and its components-tumor pT stages associations. Similar results could also be found in terms of tumor pN stages (**Supplementary Table 3**) and tumor size (**Supplementary Table 4**).

**Table 4:** Adjusted ORs and 95% CIs for the association between MetS and its components and pT stage, SEER-Medicare.

	Advanced T stage (n = 1,245)		OR (95% CI)^a,‡^	Non-advanced T stage (n = 2,540)		OR (95% CI)^a,‡^	OR (95% CI)^a,†^
	n	%		n	%		
Metabolic conditions							
Impaired fasting glucose	342	27.5	1.431 (1.263, 1.622)	695	27.4	1.301 (1.191, 1.424)	1.101 (0.944–1.282)
High blood pressure	824	65.9	1.220 (1.086, 1.371)	1663	63.4	1.079 (1.006, 1.144)	1.130 (0.980–1.304)
Elevated waist circumference/central adiposity	87	7.0	2.072 (1.663, 2.578)	167	5.7	1.667 (1.409, 1.978)	1.240 (0.944–1.635)
Low HDL cholesterol	19	1.5	1.394 (1.038, 1.759)	38	1.5	1.366 (1.025, 1.713)	1.021 (0.587–1.776)
High triglycerides	451	36.2	1.306 (1.164, 1.466)	995	36.8	1.341 (1.236, 1.452)	0.975 (0.851–1.120)
Metabolic syndrome							
NCEP-III	207	16.6	1.717 (1.478, 1.995)	440	17.0	1.765 (1.589, 1.960)	0.972 (0.811–1.165)
IDF	66	5.3	1.942 (1.513, 2.494)	135	5.2	1.903 (1.595, 2.270)	1.022 (0.755–1.382)

^a^ORs adjusted for time of diagnosis date, age at diagnosis, gender, race, registry area, household income, drug use, Medicare/Medicaid dual enrollment, time of enrollment in Medicare, tobacco and alcohol use.

^†^Comparison between advanced T stage and non-advanced T stage in the UTUC cohort.

^‡^Comparison between control group and advanced T stage UTUC or non-advanced T stage UTUC.

## Discussion

In this large population-based study, we reported an increased risk of UTUC related to MetS (defined by NCEP-III and IDF criteria). The individual components of MetS, including impaired fasting glucose, high blood pressure, elevated waist circumference/central adiposity, low HDL cholesterol, and high triglycerides were also associated with increased risks of UTUC. Furthermore, consistent associations could also be observed in the subgroup analyses by tumor grades, tumor stages (pT and pN stages), and tumor size. The time trends analysis of both MetS and its components validated the acceleration of MetS in UTUC cases and controls and the dose-response effect analysis indicated that presence of one to five of multiple MetS components, may contribute to significantly gradual increase in UTUC rates. To the best of our knowledge, this is the first study to investigate whether MetS or its components were associated with the risk of UTUC. However, due to the unavailable confounders and the limitations of obtaining data from databases, the findings should be interpreted with caution.

In the study, 647 (17.1%) patients in UTUC group and 19,755 (10.4%) of the controls met the NCEP-ATP III criteria for MetS. The proportion is comparable with other SEER-Medicare studies but is significantly lower than the contemporaneous prevalence of 25% to 34% reported by the 2012 National Health and Nutrition Examination Survey (NHANES) (13, 18, 19). The reason might be mainly attributed to the under-reporting of hypertriglyceridemia in the Medicare claims. The rate of the diagnosis was 1.5% and 1.1% in cases and controls, respectively, which were dramatically lower than the prevalence of NHANES (25% to 29%). Therefore, the main contributors to the definition of MetS in the study were impaired fasting glucose/diabetes mellitus, high triglycerides, elevated waist circumference/central adiposity, and hypertension. Using ICD-9 codes to identify MetS and its components based on claims data may contribute mistakes on classification can also be another one explanation for this. Moreover, due to the selected population consisted of subjects aged over 65 years, a real prevalence of MetS in the whole population could not be derived from the study. The under-reporting bias and selection bias should be considered when interpreting these findings.

In the study, we adopted Venn diagram and trend analysis to clearly illustrate the distribution of MetS and its components. EAPC was also calculated and it showed that the rates of MetS (NCEP-ATP III criterion) and the rates of its individual components increased over time in both UTUC and control cohorts. These findings were consistent with a previous study (20) and emphasized the contemporary dilemma caused by the swift increase of MetS. The associations between MetS and increased risks of urological tumors such as bladder cancer, prostate cancer had been reported widely (21–23). Similarly, the results in our study revealed that MetS was an independent risk factor for developing UTUC. However, based on current evidence the relationships between MetS and risks of different types of cancers weren’t consistent. In ovarian and fallopian tube cancer, and head and neck squamous cell carcinoma, inverse associations between cancer and MetS were observed (24, 25), while in studies investigating the associations between hepatocellular carcinoma, urological tumors, and esophageal adenocarcinoma and MetS, positive links were observed (7, 10, 15, 23). Moreover, even though within the same cancer type, some controversies also existed, such as the head and neck squamous cell carcinoma (24, 26). Possible reasons for the inconsistency might be attributed to variances in the adjustment of established risks, for example, tobacco use and alcohol intake, and differences in study design. In the study, three methods were adopted to reduce the possible biases: Firstly, we selected eligible UTUC cases, and then the controls were generated randomly and matched with cases through diagnosis date and pseudo-diagnosis date to minimize the effect of possible diagnostic trends. Secondly, in the logistic regression analyses, we adjusted both the sociodemographic information and tobacco and alcohol use to reach relatively precise results. Thirdly, all the SEER-Medicare codes in the study had been validated and used in previous studies (10, 11). Notably, given the nature of case-control study, we did not calculate the population attributable risk (PAR).

As described by previous studies, elevated waist circumference/central adiposity, a major component of MetS, had the strongest association with endometrial cancers (11, 27), and it was believed that elevated waist circumference/central adiposity was associated with worse outcomes in several cancers including UTUC (12, 23, 28). In line with these studies, results in our study indicated that elevated waist circumference/central adiposity is the strongest risk factor for UTUC among MetS components. This can also be observed in the subgroup analysis by tumor grades, tumor stages, and tumor size. Notably, we found increased risks of UTUC associated with MetS and its component factors with no observed heterogeneity in OR estimates. Results on this in UTUC corroborate previous researches on bladder cancer and endometrial cancer (11, 23). Moreover, we found no significant results in the multivariate analysis of MetS and tumor grades, tumor stages, and tumor size within the UTUC cohort, a possible explanation for this was MetS was only associated with an increased risk of UTUC, while it had no relationship with the severity of UTUC, which may directly be affected by the tumor biology. Similar findings were only reported in endometrial cancer (11, 29). Further well-designed studies are required to verify this finding in UTUC cases.

Using dose-response effect analysis, we found that risk of UTUC increased with the growing number of simultaneously existed MetS components. The most important observation is that even the presence of only one MetS component is still associated with an elevated risk of developing UTUC. Thus, although many patients do not yet meet the diagnostic criterion of MetS, they still should pay attention to the control of the individual component, which may lower the possibility of developing not only UTUC but also several other kinds of tumors.

With the large sample size of the study, we also tested the associations between potential factors (mainly cigarette intake or alcohol use) and both MetS and UTUC (shown in **Supplementary Tables 5**-**6**). Results of multivariate analysis indicated that alcohol or tobacco use was related to elevated risks of developing MetS in the whole population (OR: 1.303, 95% CI: 1.052–1.552; OR: 1.472, 95% CI: 1.256–1.688, respectively). In the multivariate analysis without adjusting for gender and established risk factors for UTUC, we verified that MetS and its components were still associated with increased risk for UTUC (ORs ranged from 1.102 to 2.093).

Although a number of articles revealed the associations between MetS and its components with tumors, the mechanisms that linked MetS and cancer risk were not yet clearly characterized. Some possibilities were: (A) Patients with MetS tend to have high levels of cholesterol, which could stimulate the proliferation of epithelial cells. And high cholesterol levels correlate with high levels of vascular endothelial growth factor (VEGF) in plasma. Both of these two stimulate proliferation of epithelial cells (30). (B) Moreover, MetS patients tend to have high levels of adipose tissue, which could secrete leptin and it has been validated that leptin could also enhance angiogenesis (31). In insulin resistance, insulin and insulin-like growth factors can also significantly improve cancer development with proliferation promotion and antiapoptotic impacts (32). (C) Obesity, especially the central obesity, has been indicated as a factor of MetS and cancer carcinogenesis (33). Moreover, obesity status is associated with insulin resistance, higher blood free fatty acids, and chronic micro-inflammatory status, which mediated and affected by several pro-inflammatory cytokines, such as C-reactive protein (CRP) and tumor necrosis factor-α (34). These molecules can also promote tumor development and suppress immune response. With all these synergic effects, no doubt that we found the strongest strength of association in central obesity and risk of UTUC. (D) Furthermore, the association between adiposity and lower mitochondrial function, and therefore circulating reactive oxygen species (ROS) accumulate dramatically, which can also cause damage to DNA (35).

Our findings may have some implications for basic and clinical researches. Firstly, the establishment of MetS and its components as independent risk factors for UTUC could provide rationality to studies pursuing novel pharmacological roles beyond classical effects. In the past, many drugs, such as statins and metformin used to treat the components of MetS in cancer patients, have been proved to improve the cancer-specific survival significantly. Recently, several studies have also proved the cancer prevention effect of these drugs (36). However, there were no studies on the topic reported in UTUC. Therefore, the finding that MetS could increase the risk of developing UTUC could bring new insights into the prevention of UTUC and it will be a clinically available way to prevent patients with MetS from UTUC. Secondly, when it comes to the study in oncology, non-tumor factors like metabolic syndrome, vitamin intake, and medication use, etc. might also be associated with cancer risks. Thirdly, the potential mechanisms implied in the study are required to be verified in basic science.

Some potential residual confounders should be discussed. Firstly, the alcohol and cigarette intake, which were very important risk factors for several cancers and were always under-reported and under-rated. Additionally, there was no available data on the duration or the amount of intake and we could not derive ever-use data from claims data. The limitation might be more pronounced for alcohol factors, because of only 4.8% of the cases and 1.3% of the controls were identified as alcohol users. Currently, there was no data on the proportion of ever-consumers in UTUC cases. A similar problem was also evident when it came to occupational exposures, such as radiation, which were also regarded as an important risk factor for UTUC. The concern on this must be considered and further studies are expected to investigate the issue better. Secondly, physical inactivity and dietary intake. The definition of inactivity here is a contraction of body or little exercise that reduces energy expenditure below the basal level. A previous review had well-described the relationship between diet and physical activity and cancers (37). It indicated that physical activity was inversely related to increased risks of developing many cancers and diets like fibers, fruits, and vegetables could significantly reduce the cancer incidence, especially in children aged less than 8 years. Mechanisms on this might be similar as described above and mainly involve these items: systematic inflammation, DNA damage, stress response, and imbalance of homeostasis, etc. In the study, factors on this were not available and could not be retrieved, which might lead to the bias and overestimated results. Thirdly, in the study we considered the medication use as a potential confounder that may affect the estimated ORs, while participants may be diagnosed with hypertension, dyslipidemia, etc before their enrollment to Medicare and some may have already received relevant treatments. Based on the nature of the SEER-Medicare linked data, information on this was difficult to be ascertained. Given the above concerns, our results should be interpreted with caution.

The strengths of the study are related to the population source, definitions of case and control, as well as the mutual adjustment for analysis. The large sample size was obtained from SEER registries, in which the data was ascertained annually with quality control checks and SEER-Medicare is an excellent resource for studying rare cancers like UTUC. Additionally, the SEER database was selected to be highly representative of the real situation, therefore the findings in the study could be generalized to the general population aged over 65 years. However, several limitations existed: Firstly, although the identification of metabolic factors based on Medicare claims data avoid recall bias related to personal interview measurements, under-ascertainment of some exposures is inevitable, such as doses of cigarette and tobacco use, and chemicals exposure, etc. This is also common in previously published studies (10, 11). However, both the MetS and these factors were potentially underrated, the possible biases thus may not influence the ORs. Secondly, the generalization of the findings to the general population could be restricted by the higher proportion of White people included in the SEER registries. Whether the race difference could affect the association was unclear yet. Thirdly, actual values for BMI, blood pressure, fasting glucose, and cholesterol are presumably unavailable in this database, limiting the analysis to dichotomous categorizations, and limiting the ability to fully examine these exposures and their associations with UTUC. Last but not the least, using ICD-9 codes to identify MetS and its components based on claims data may contribute to mistakes on classification. While based on several studies with similar methodology reporting the associations between MetS and many cancers epidemiology, especially the urothelial cancer of the bladder, which has great similarity with UTUC, the study thus can be a tentative on the topic until the findings were validated by more studies.

In conclusion, the results of this population-based study indicated that MetS and its components were significant risk factors for UTUC. Consistent associations could also be observed in the subgroup analyses by tumor stages, grades and size. Even the presence of only one MetS component is still associated with an elevated risk of developing UTUC. Thus, approaches to control the epidemic of MetS and its components may contribute to a reduction in the UTUC burden. Although this is the first study investigating the relationship between MetS and the risk of UTUC with intrinsic limitations, the findings should be considered tentative until ascertained by more researches.

## Data Availability Statement

The original contributions presented in the study are included in the article/**Supplementary Material**; further inquiries can be directed to the corresponding authors.

## Ethics Statement

Ethical review and approval was not required for the study on human participants in accordance with the local legislation and institutional requirements. Written informed consent for participation was not required for this study in accordance with the national legislation and the institutional requirements.

## Author Contributions

YL, WZ, and XQL conceived and designed the study. XL provided administrative support. YL, WZ, and SF collected and assembled the data. XQL, YL, SJF, and YXS analyzed and interpreted the data. All authors wrote the manuscript. All authors contributed to the article and approved the submitted version.

## Funding

This work was supported by the Zhao Yi-Cheng Medical Science Foundation, China (grant no. ZYYFY2018031).

## Conflict of Interest

The authors declare that the research was conducted in the absence of any commercial or financial relationships that could be construed as a potential conflict of interest.

## Abbreviations

UTUC, upper tract urothelial carcinoma; MetS, metabolic syndrome; SEER, Surveillance, Epidemiology and End Results; NCEP-III, National Cholesterol Education Program Adult Treatment Panel III; IDF, International Diabetes Foundation; EAPC, estimated annual percentage changes; RNU, radical nephroureterectomy; ICD, International Classification of Diseases; HMO. health maintenance organization; CCI, Charlson comorbidity index; SD, standard deviation; ORs, odds ratios; Cis, confidence intervals; IFG, impaired fasting glucose; HBP, high blood pressure; EWC/CA, elevated waist circumference/central adiposity; HDL, high-density lipoprotein; L-HDLC, low high-density lipoprotein cholesterol; HTG, high triglycerides.
