# Supplementary material for: Metabolic Syndrome and Risk of Upper Tract Urothelial Carcinoma: A Case-Control Study From Surveillance, Epidemiology and End Results-Medicare-Linked Database
Source: Front Oncol. 2021 Jan 21;10:613366. doi: 10.3389/fonc.2020.613366 (PMC7859618; doi:10.3389/fonc.2020.613366)
Supplement: Supplementary file 1 [file DataSheet_1.docx]

Supplementary Material

# Supplementary Tables

| **Supplementary Table 1** Medicare claims data code definitions used to define metabolic syndrome factors. | |
| --- | --- |
| Factor | Code-based definitions |
| Central adiposity | A diagnosis of central adiposity (specifically ICD-9: 278, 278.0, 278.00, 278.01, 278.02, 278.03, 278.1, V77.8^a^) |
| Impaired fasting glucose | A diagnosis of type 2 diabetes or impaired fasting glucose (ICD-9: 250.X0, 250.X2, 790.2, 790.21, 790.22, 790.29)^b^ |
| High blood pressure | A diagnosis of hypertensive disease (ICD-9: 401-405) |
| Low HDL cholesterol | A diagnosis of lipoprotein deficiency (ICD-9: 272.5, 272.5X) |
| High triglycerides | A diagnosis of pure hyperglycemia, mixed hyperlipidemia, hyperchylomicronemia, or other unspecified hyperlipidemia (ICD-9: 272.1, 272.1X, 272.2, 272.2X, 272.3, 272.3X, 272.4, 272.4X)^c^ |
| Dysmetabolic syndrome | A diagnosis of metabolic syndrome (ICD-9: 277.7, started use in 2001) |

^a^Code for central adiposity (V77.8) only available from 2001-2007

^b^Codes 250, 250.X were not included because they generically reference both type 1 and 2 diabetes, and codes 250.X1 and 250.X3 were not included because they also include type 1 diabetes

^c^Codes 272, 272.0, 272.0X are not included

**Supplementary Table 2** ORs and 95% CIs for rates of UTUC according to number of metabolic syndrome components.

| Items | OR (95% CI) | *P*-value |
| --- | --- | --- |
| One vs. zero components | 1.600 (1.437-1.782) | **0.000** |
| Two vs. zero components | 2.152 (1.715-2.590) | **0.000** |
| Three vs. zero components | 2.530 (2.218-2.886) | **0.000** |
| Four vs. zero components | 2.844 (2.455-3.335) | **0.000** |
| Five vs. zero components | 4.292 (3.166-5.418) | **0.010** |
| *P* for trend ^a^ | — | **0.000** |

^a^ Tested by *χ²* test.

**Supplementary Table 3** Adjusted ORs and 95% CIs for the association between MetS and its components and pN stage, SEER-Medicare

|  | pN+ (n=390) | | OR (95%CI)^a^ | pN0 (n=3,395) | | OR (95%CI)^a^ | OR (95%CI)^a, †^ |
| --- | --- | --- | --- | --- | --- | --- | --- |
|  | n | % |  | n | % |  |  |
| Metabolic conditions |  |  |  |  |  |  |  |
| Impaired fasting glucose | 102 | 26.1 | 1.339 (1.069, 1.679) | 935 | 25.8 | 1.316 (1.218, 1.422) | 1.018 (0.802, 1.293) |
| High blood pressure | 256 | 65.7 | 1.165 (1.022, 1.302) | 2231 | 64.4 | 1.126 (1.050, 1.207) | 1.057 (0.847, 1.316) |
| elevated waist circumference/central adiposity | 34 | 8.6 | 2.627 (1.846, 3.741) | 220 | 7.1 | 2.107 (1.844, 2.407) | 1.250 (0.859, 1.819) |
| Low HDL cholesterol | 8 | 2.0 | 1.883 (1.023, 2.741) | 49 | 1.8 | 1.643 (1.271, 2.125) | 1.144 (0.545, 2.407) |
| High triglycerides | 141 | 36.2 | 1.302 (1.058, 1.601) | 1305 | 36.4 | 1.317 (1.227, 1.413) | 0.989 (0.795, 1.231) |
| Metabolic syndrome |  |  |  |  |  |  |  |
| NCEP-III | 69 | 17.8 | 1.851 (1.427, 2.401) | 578 | 17.2 | 1.790 (1.635, 1.959) | 1.035 (0.786, 1.362) |
| IDF | 21 | 5.5 | 1.974 (1.271, 3.067) | 180 | 5.4 | 1.976 (1.700, 2.302) | 0.998 (0.627-1.589) |

^a^ ORs adjusted for time of diagnosis date, age at diagnosis, gender, race, registry area, household income, drug use, Medicare/Medicaid dual enrollment, time of enrollment in Medicare, tobacco and alcohol use.

^†^ Comparison between pN+ UTUC and pN0 UTUC in the UTUC cohort.

**Supplementary Table 4** Adjusted ORs and 95% CIs for the association between MetS and its components and tumor size, SEER-Medicare

|  | > 3 cm (n=1,546) | | OR (95%CI)^a^ | ≤ 3 cm (n=2,239) | | OR (95%CI)^a^ | OR (95%CI)^a, †^ |
| --- | --- | --- | --- | --- | --- | --- | --- |
|  | n | % |  | n | % |  |  |
| Metabolic conditions |  |  |  |  |  |  |  |
| Impaired fasting glucose | 397 | 25.7 | 1.301 (1.163, 1.460) | 640 | 26.2 | 1.342 (1.223, 1.478) | 0.952 (0.820, 1.107) |
| High blood pressure | 1006 | 65.1 | 1.161 (1.045, 1.289) | 1481 | 64.5 | 1.133 (1.038, 1.234) | 1.025 (0.892, 1.174) |
| elevated waist circumference/central adiposity | 122 | 7.9 | 2.362 (1.961, 2.842) | 132 | 7.0 | 2.078 (1.766, 2.452) | 1.133 (0.882, 1.450) |
| Low HDL cholesterol | 26 | 1.7 | 1.123 (0.975, 1.271) | 31 | 1.8 | 1.265 (0.883, 1.804) | 0.889 (0.526, 1.503) |
| High triglycerides | 566 | 36.6 | 1.328 (1.195, 1.472) | 880 | 36.2 | 1.306 (1.195, 1.426) | 1.017 (0.889, 1.163) |
| Metabolic syndrome |  |  |  |  |  |  |  |
| NCEP-III | 278 | 18.2 | 1.889 (1.658, 2.151) | 369 | 17.4 | 1.817 (1.626, 2.028) | 1.039 (0.877, 1.233) |
| IDF | 80 | 5.2 | 1.894 (1.510, 2.375) | 121 | 4.6 | 1.675 (1.371, 2.043) | 1.132 (0.837-1.529) |

^a^ ORs adjusted for time of diagnosis date, age at diagnosis, gender, race, registry area, household income, drug use, Medicare/Medicaid dual enrollment, time of enrollment in Medicare, tobacco and alcohol use.

^†^ Comparison between UTUC (> 3 cm) and UTUC (≤ 3 cm) in the UTUC cohort.

**Supplementary Table 5** Univariable analysis and multivariate analysis for the association between MetS and potential confounders in the whole cohort.

|  | Univariate analysis | Multivariate analysis ^a^ |
| --- | --- | --- |
| Age | 1.013 (0.876, 1.149) | 0.976 (0.783, 1.149) |
| Gender (Male/Female) | 1.132 (0.926, 1.337) | 1.055 (0.901, 1.211) |
| Tobacco use (Yes/No) | 1.547 (1.224, 1.871) | 1.472 (1.256, 1.688) |
| Alcohol use (Yes/No) | 1.368 (1.175, 1.560) | 1.303 (1.052, 1.552) |

^a^ Adjusted for time of diagnosis date, race, registry area, household income, drug use, Medicare/Medicaid dual enrollment, and time of enrollment in Medicare.

**Supplementary Table 6** Adjusted ORs and 95% CIs for the association between MetS and its components and UTUC, SEER-Medicare

|  | OR (95%CI)^a^ |
| --- | --- |
|  |  |
| Metabolic conditions |  |
| Impaired fasting glucose | 1.292 (1.123, 1.461) |
| High blood pressure | 1.264 (1.231, 1.297) |
| elevated waist circumference/central adiposity | 1.832 (1.664, 2.002) |
| Low HDL cholesterol | 1.339 (1.102, 1.577) |
| High triglycerides | 1.255 (1.217, 1.294) |
| Metabolic syndrome |  |
| NCEP-III | 1.292 (1.237, 1.364) |
| IDF | 1.865 (1.687, 2.093) |
| Age | 1.025 (0.836, 1.214) |
| Gender (Male/Female) | 0.855 (0.604-1.134) |
| Tobacco use (Yes/No) | 1.768 (1.256-2.231) |
| Alcohol use (Yes/No) | 1.483 (1.120-2.192) |

^a^ Adjusted for time of diagnosis date, race, registry area, household income, drug use, Medicare/Medicaid dual enrollment, and time of enrollment in Medicare.

# Supplementary Figures
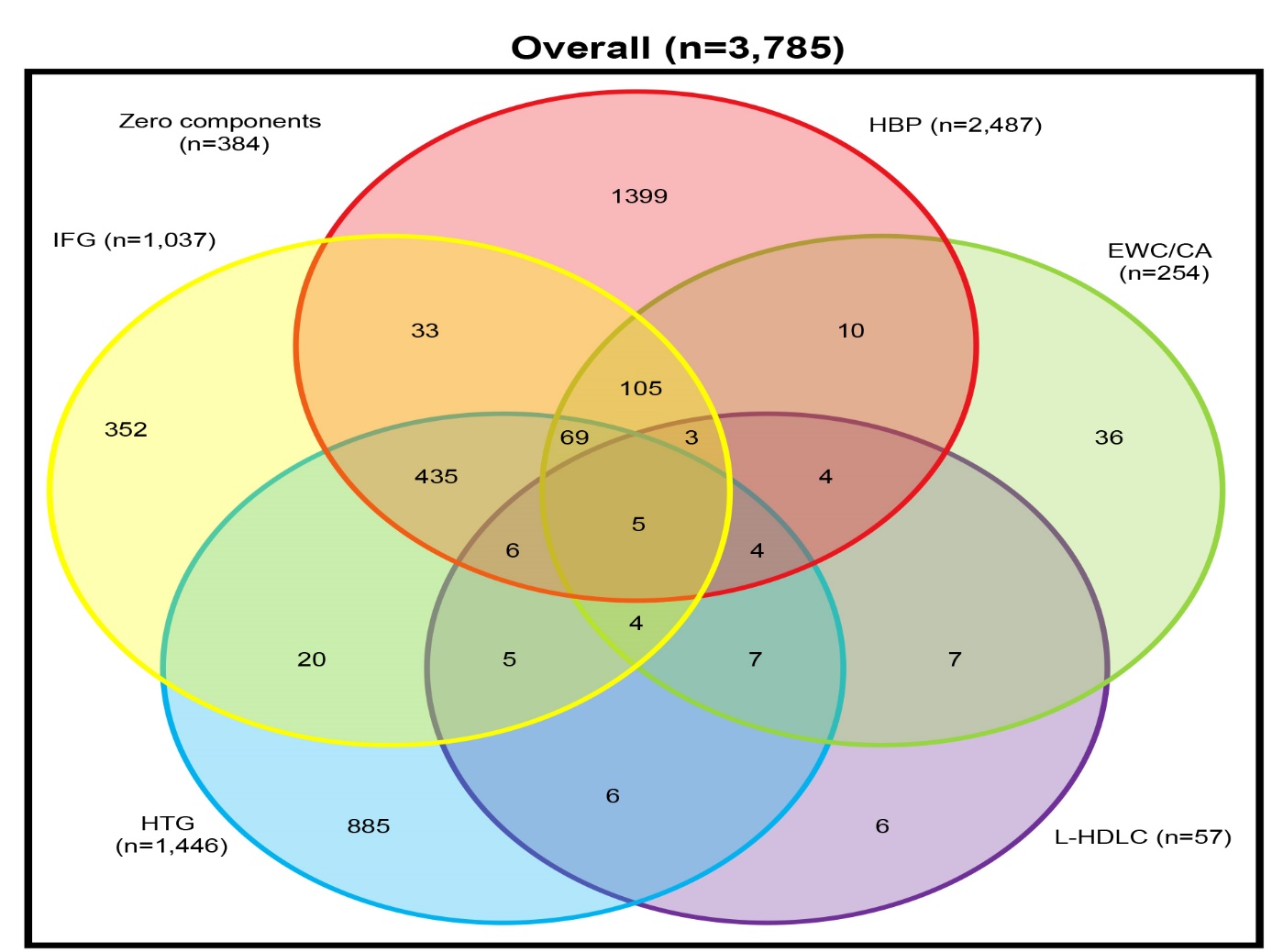


# Supplementary Figure 1. Venn diagram to show the MetS components distribution in UTUC cohort.


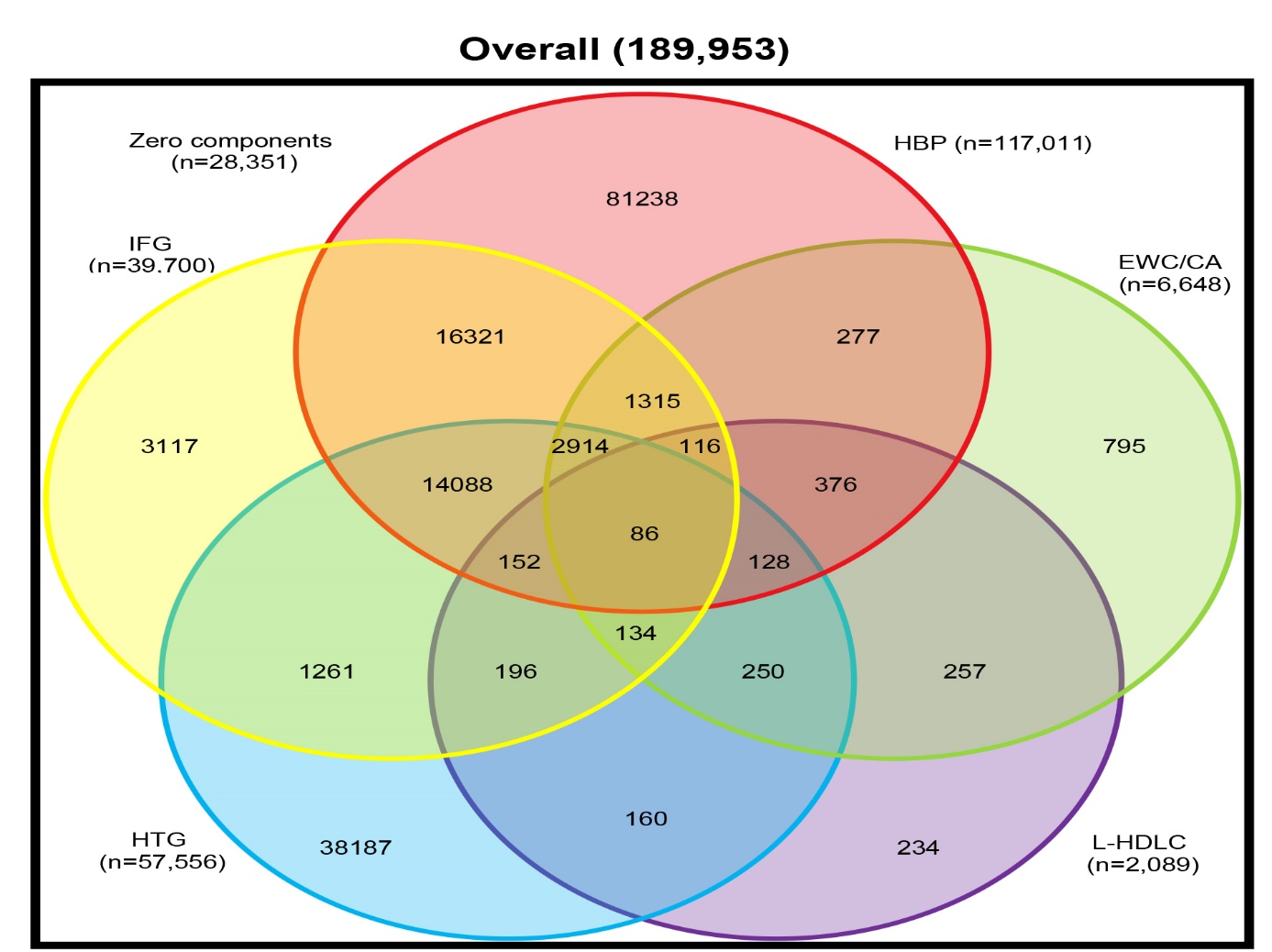


**Supplementary Figure 2.** Venn diagram to show the MetS components distribution in the control cohort.


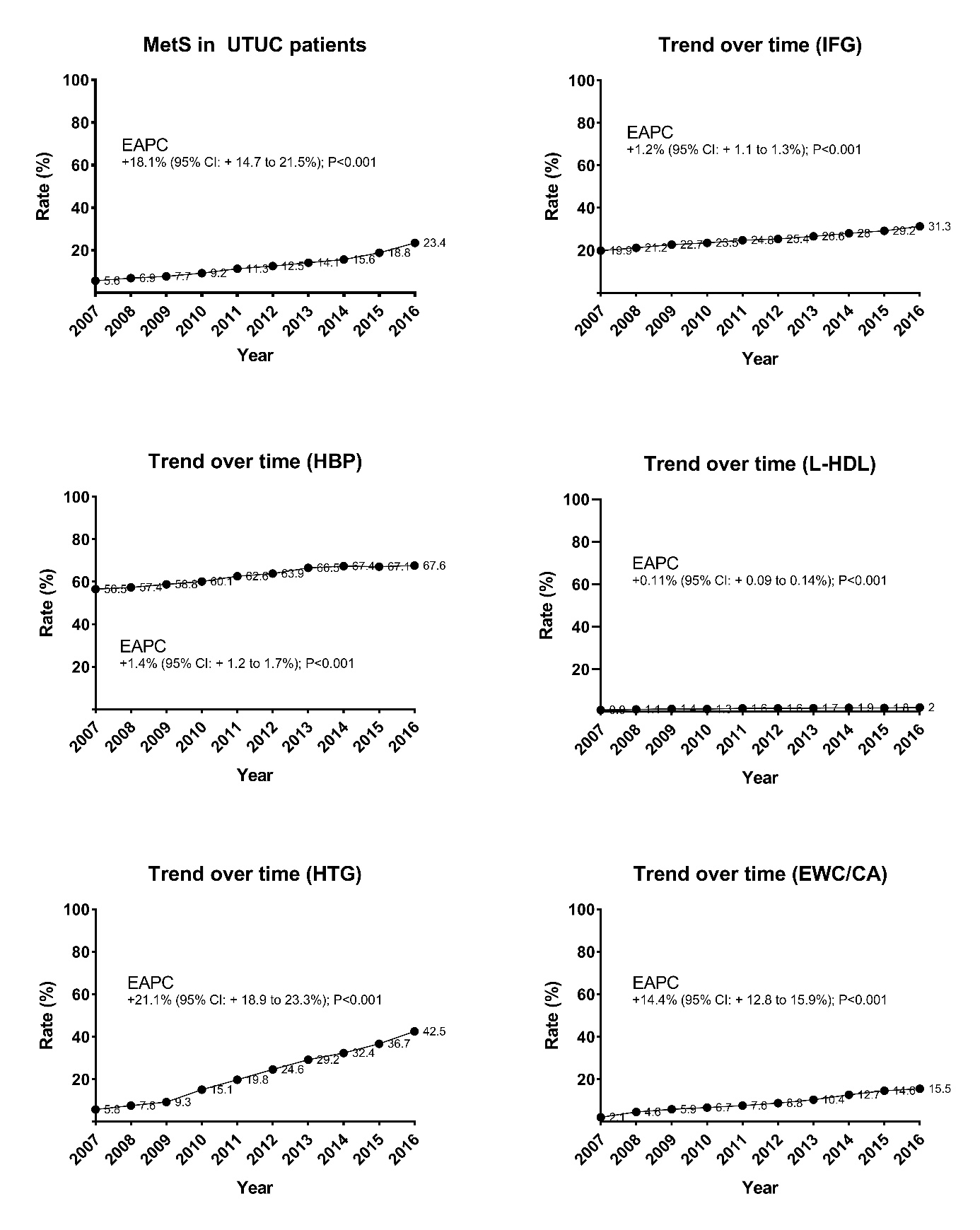


**Supplementary Figure 3.** Time trends to show the rates of MetS and its components in the UTUC cohort.


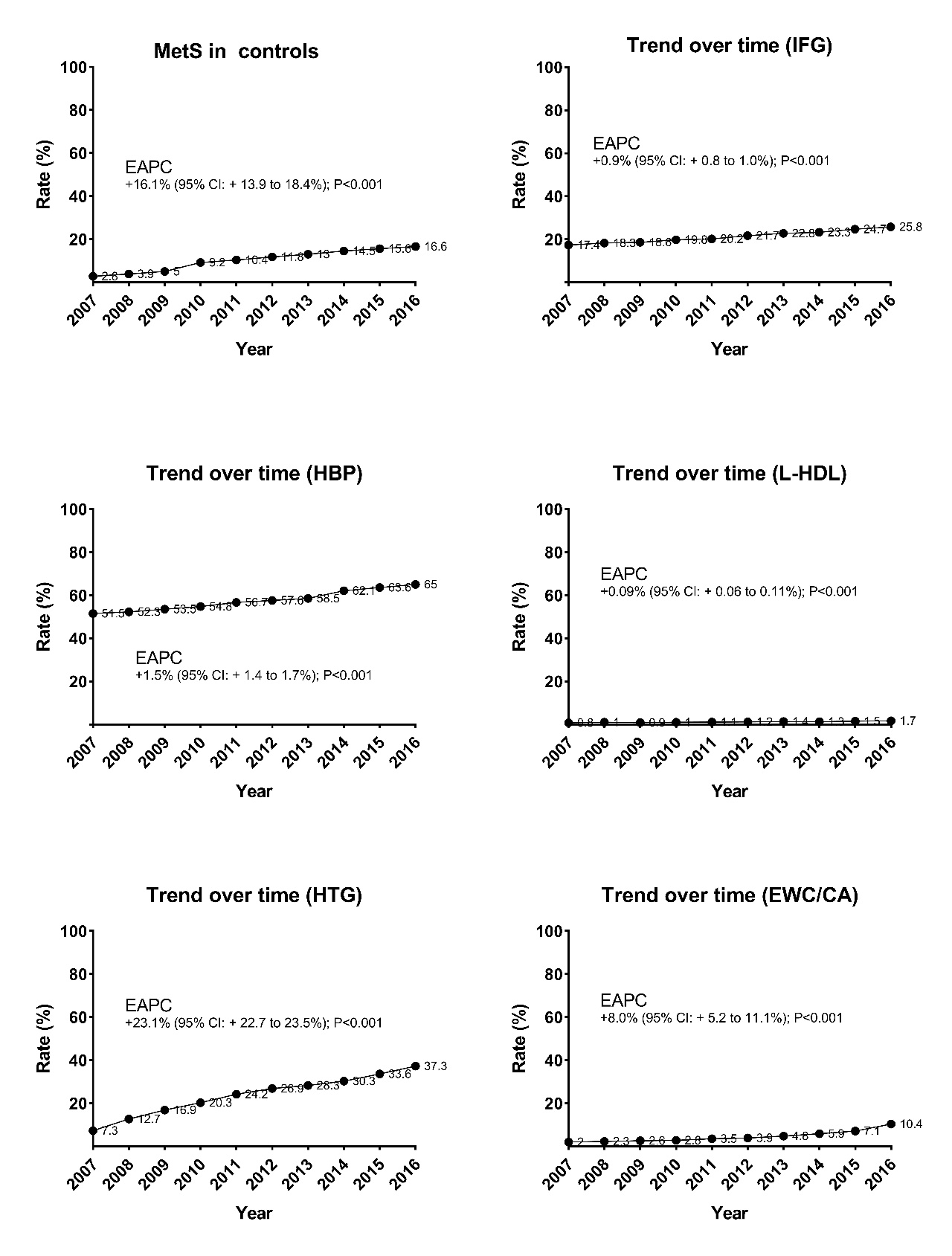


**Supplementary Figure 4.** Time trends to show the rates of MetS and its components in the control cohort.
